# Forging New Therapeutic Targets: Efforts of Tumor Derived Exosomes to Prepare the Pre-Metastatic Niche for Cancer Cell Dissemination and Dormancy

**DOI:** 10.3390/biomedicines11061614

**Published:** 2023-06-01

**Authors:** Ranvir Bhatia, Joanna Chang, Jessian L. Munoz, Nykia D. Walker

**Affiliations:** 1 Perelman School of Medicine, University of Pennsylvania, Philadelphia, PA 19104, USA; ranvir.bhatia@pennmedicine.upenn.edu; 2 Department of Biological Sciences, University of Maryland, Baltimore, MD 21250, USA; 3 Division of Perinatal Surgery, Texas Children’s Hospital, Houston, TX 77030, USA; jessianmunoz@gmail.com; 4 Division of Maternal Fetal Medicine, Baylor College of Medicine, Houston, TX 77030, USA

**Keywords:** cancer metastases, tumor derived extracellular vesicles, nanocarriers, drug delivery systems, mesenchymal stem cells, pre-metastatic niche, cancer stem cells, organotropism, lymphatics, cellular dormancy, and liquid biopsy

## Abstract

Tumor-derived exosomes play a multifaceted role in preparing the pre-metastatic niche, promoting cancer dissemination, and regulating cancer cell dormancy. A brief review of three types of cells implicated in metastasis and an overview of other types of extracellular vesicles related to metastasis are described. A central focus of this review is on how exosomes influence cancer progression throughout metastatic disease. Exosomes are crucial mediators of intercellular communication by transferring their cargo to recipient cells, modulating their behavior, and promoting tumor pro-gression. First, their functional role in cancer cell dissemination in the peripheral blood by facilitating the establishment of a pro-angiogenic and pro-inflammatory niche is described during organotro-pism and in lymphatic-mediated metastasis. Second, tumor-derived exosomes can transfer molecular signals that induce cell cycle arrest, dormancy, and survival pathways in disseminated cells, promoting a dormant state are reviewed. Third, several studies highlight exosome involvement in maintaining cellular dormancy in the bone marrow endosteum. Finally, the clinical implications of exosomes as biomarkers or diagnostic tools for cancer progression are also outlined. Understanding the complex interplay between tumor-derived exosomes and the pre-metastatic niche is crucial for developing novel therapeutic strategies to target metastasis and prevent cancer recurrence. To that end, several examples of how exosomes or other nanocarriers are used as a drug delivery system to inhibit cancer metastasis are discussed. Strategies are discussed to alter exosome cargo content for better loading capacity or direct cell targeting by integrins. Further, pre-clinical models or Phase I clinical trials implementing exosomes or other nanocarriers to attack metastatic cancer cells are highlighted.

## 1. Tumor-Derived Exosomes (TD-EVs) Overview

**Figure 1:**
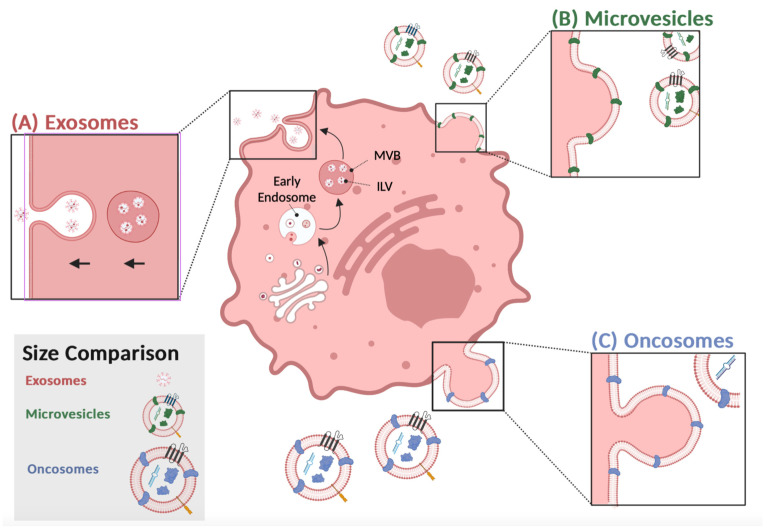
Tumor-derived extracellular vesicles release: (**A**) Proteins from the membrane, cytosol, or the endomembrane system invaginate in an early endosome. Late endosomes, or multivesicular bodies (MVB), sort proteins in intraluminal vesicles (ILV). Once MVBs fuse with the plasma membrane and release exosomes into the extracellular space. (**B**) Microvesicles (MV) bleb directly from the membrane, adapting a portion of the parent cell. Microvesicles are released through outward budding. (C) Oncosomes are released through pinched blebbing. The surface proteins and nucleic acids of different colors depict the unique cargo of MV and oncosomes. Proteins of the same color depict overlaps between the vesicles. This figure was created using BioRender.com.

In the endosome compartment, exosome synthesis occurs when multivesicular bodies mature into intraluminal vesicles (Figure 1A). Fusing intraluminal vesicles with a plasma membrane releases exosomes into the extracellular space [1,2]. Exosomes can be retrieved by endocytosis or receptor-mediated uptake, suggesting a selective intercellular communication between the donor and recipient cells [3,4]. Exosomes released from donor cells contain nucleic acids or proteins, which appear to be strategically used to modify the recipient cell’s function in a way that benefits the donor cell [5]. Thus, isolating exosomes, identifying their cargo and their intended target cell are potential biomarkers, diagnostic tools or therapeutic targets for disease progression [5,6]. In healthy cells, exosomes help to maintain normal physiology and are a type of intercellular communication system used mainly by immune cells [5,7]. However, the number of exosomes secreted by cancer cells exceeds those of healthy cells, especially during oncogenesis and tumor suppression [8,9]. The type of exosome cargo isolated from tumor cells consists of mainly nucleic acids associated with RNA processing, including microRNA (miRs), long-noncoding RNAs, and circular RNAs, which are useful cancer biomarkers that are distinguishable from those released from noncancerous cells [10,11,12]. Recent studies, using organoids from colon cancer cells, identified two distinct populations of exosomes, and both were enriched with a unique set of proteins with specific functions that supported colon cancer progression [13], suggesting that exosomes are heterogeneous, which means that their composition and function will differ, as well as what cells are targeted and how they are altered. Developing targeted therapeutic approaches requires studying these interactions and understanding what contributes to exosome heterogeneity within tumors.

By secreting exosomes into the circulation, tumor cells can communicate with noncancerous cells at distant sites in preparation for tumor dissemination in an autocrine-, paracrine-, or endocrine-like manner [14]. In the bone marrow, exosomes can induce cell-to-cell interactions via gap juxtracrine communication to facilitate cellular dormancy [15,16]. Since exosomes regularly migrate through the circulatory system as extracellular messengers, it is advantageous for these vesicles to contain cholesterol, sphingomyelin, and ganglioside GM3, which act as protective proteins against the complement system, preventing degradation while in circulation [17]. Regarding tumor progression, exosomes promote metastasis by coordinating communication between tumor cells and endothelial or immune cells [18]. Exosomes derived from colon cancer, for example, regulate the vascular volume by stimulating angiogenesis and altering the cellular permeability by targeting *KLF2* and *KLF4* [19]. Another example in renal carcinoma, CD105+ CSC-derived exosomes promoted endothelial cell vascular differentiation and proliferation through proangiogenic miRs and mRNA transfer [20]. 

### TD-EVs Involvement in Metastatic Disease

Tumor-derived exosomes are a type of extracellular vesicle distinguished from apoptotic bodies, microvesicles, and oncosomes by their size, morphology, and protein markers [21,22,23,24]. Similar to exosomes, tumor cells secrete microvesicles into the extracellular space, but they are called ectosomes, since their formation involves the outward budding of the plasma membrane (Figure 1B) [25]. They are secreted from the plasma membrane and can transfer cytoskeletal and microtubule proteins by autologous communication with cells within the tumor microenvironment to facilitate proliferation [26]. In addition, they can also stimulate adjacent cells by transferring proteins that activate oncogenic signaling cascades to induce cell invasion. 

**Figure 2:**
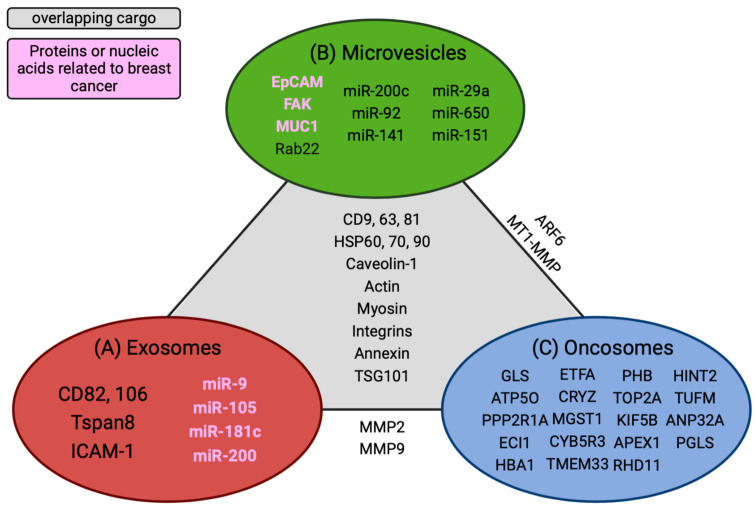
Overlapping cargo comparisons between extracellular vesicles. Unique nucleic acids and proteins are grouped within each specific vesicle type. (**A**) Identified exosome cargo. (**B**) Microvesicle cargo. (**C**) Common oncosome cargo. Overlapping cargos that are common among all EVs are delineated in the gray section of the diagram. Cargos found between two EVs are listed in tangent with the solid line that connects them. Biomarkers or nucleic acids highlighted in pink are associated with breast cancer. This figure was created using BioRender.com.

Furthermore, microvesicles have been isolated from the peripheral blood of cancer patients, suggesting that they can promote long-distance communication to influence metastatic spread or aid in preparing the recruitment of the pre-metastatic niche [27,28]. According to Muralidharan-Chari et al., *ARF6* regulates the sorting and shedding of microvesicles from tumor cells at specific regions of the plasma membrane. Microvesicles are directly transferred to the plasma membrane of the recipient cell, altering their function to promote cell growth [29]. In contrast, exosomes bind to their target cells and release their contents internally, activating signaling pathways and altering gene expressions in those cells [30]. Numerous studies have described overlapping markers that are shared between microvesicles and exosomes. In contrast, the proteomics analysis of microvesicle cargo was similar to that of the plasma membrane of the donor cell, whereas exosome biogenesis or cargo was synthesized in the endosomes (Figure 2) [27]. 

Large oncosomes (LO) are another type of extracellular vesicle worthy of attention for their involvement in tumor progression. They are larger (1–10 μm) compared to microvesicles or exosomes, are secreted by tumor cells, and transport proteins from the plasma membrane to recipient cells [25] (Figure 1C). As a result of membrane blebbing, membrane proteins from the donor cell are transferred to the recipient cell, mimicking the donor’s physiological state. Oncosomes are a source of oncogenic material excreted by metastatic cells and often have similar EV-associated markers, such as CD9, and CD81 is enriched in LOs but to a lesser degree than exosomes or microvesicles [31]. In addition, they contain signaling factors, RNA processing molecules, or growth factors related to tumor progression [32]. Anaglous to the other types of TD-EVs described above, they are more prevalent in metastatic than benign tumors, suggesting that the cargo are potential biomarkers [33]. Isolating these vesicles from the peripheral blood and analyzing their cargo will help us decipher their function throughout tumor dissemination to aid us in developing methods to inhibit their transition from the primary tumor site to distant tissue.

Oncosomes are secreted in large amounts by tumor cells, and the amount seems to correlate with aggressive tumors. Di Vizio et al. demonstrated that oncosomes are secreted in large amounts upon the silencing of a protein, Diaphanous-related formin-3 (DIAPH3), which is involved in cell motility [34]. Depending on the type of cargo, LOs can induce an amoeboid shape in the recipient cell, which is associated with cell invasion and migration [35]. For instance, prostate cancer cell overexpression of Akt also triggers the release of oncosomes, resulting in cells with amoeboid migration properties rather than mesenchymal shapes; this implies that the tumor cells likely dictate the invasive cells’ migration properties based on the type of matrix they will traverse. Conely et al. measured the mRNA expression from LOs compared to microvesicles and exosomes isolated from glioblastoma cell lines and the peripheral blood of breast cancer patients. They discovered that the mRNA cargo was the same between the TD-EVs and within the two sample sets, with 5% of the genes unique to Los associated with the plasma membrane, transporters, and receptors [36].

Overall, TD-EVs are secreted in higher amounts than normal cells. They are potential diagnostic markers based solely on the number of particles isolated in bodily fluids from cancer patients. Additionally, the three TD-EVs carry different cargos, which use other mechanisms to drive metastasis. The caveat is that tetraspanins, *ALIX*, and *TSG101* are commonly shared proteins among TD-EVs, requiring additional markers to stratify the unique features associated with the different TD-EVs. An ideal approach would be quantifying the protein markers enriched in one type of TD-EV over another and the tumor markers associated with specific vesicles. Mincaicchi et al. studied used enrichment strategies in proteomics to isolate three different TD-EVs and compared the protein expressions among them in search of distinct proteins solely expressed in LOs (Figure 2). They classified LOs as a specific type of TD-EV that influences metabolic changes in prostate cancer patients via *GOT1* uptake in recipient cells to promote cell proliferation, while exosome proteins are involved in driving cell motility and adhesion [31]. These studies provided insight into the mechanism used by tumor cells to alter naïve cell functions. Additionally, we learned that, despite having similar membrane proteins, TD-EVs invoke different functions to perpetuate metastasis and should be further investigated as potential nanomedicine strategies to alter their communication with their recipient cells.

## 2. Exosomal-Induced Metastasis via Organotropism

Metastatic sites are well established to be disease- and organ-dependent [37]; tissue tropism is likely the interactions between the cancer cells and microenvironment, especially at distant sites. Stephen Paget’s theory of seed and soil suggests that disseminating tumor cells (seed) must recognize specific organ cell entry and colonization. He speculated that breast cancer patients undergo metastasis, in which secondary outgrowths are organ-specific compared to other types of cancer [38]. Expanding on Paget’s observations, the metastatic niche model proposed by Psaila et al. suggested that organs are primed to mimic the primary tumor milieu in preparation for colonization by disseminating cancer cells to undergo colonization and fostering cellular dormancy [39,40]. A premetastatic niche consists of suppressive immune cells, a promiscuous extracellular matrix, and supporting stromal cells that attract disseminating cancer cells for colonization [39,41]. Premetastatic niches are crucial for successful tumor cell colonization and are mediated by growth factors, cytokines, and exosomes released from the primary tumor [37,42,43].

In a series of experiments, Hoshino et al. showed that breast cancer cell lines with specific tissue tropisms in the lung, liver, brain, or bone secrete exosomes with a matching biodistribution and preferential uptake at these sites [44,45]. Notably, mice pre-educated with exosomes derived from a lung trophic cell line displayed increased lung metastasis when injected with bone trophic cancer cells. These findings suggest that exosomes may be pivotal in establishing a pre-metastatic niche via organotropism. Integrins α6β1 and α6β4 were linked to lung metastasis, integrin αvβ5 to liver metastasis, and integrin β3 to brain metastasis. Altogether, the elegant work of Hoshino et al. suggested a profound phenomenon for exosome-driven cancer metastasis, suggesting that specific integrins may serve as a zip code for exosomes and directs them to the appropriate sites to deliver their cargo and reprogram the pre-metastatic niche to promote tumor cell colonization in distant sites. In addition to organotropism, exosome composition, such as its lipid moieties, might dictate their uptake and drive tumor progression in glioblastoma cells [46]. Changes in the pH within the tumor microenvironment have also been postulated to drive exosome secretion and uptake by metastatic melanoma cells, which alters the lipid composition of the cell. Additionally, an acidic tumor milieu correlates with an increased TD-EV caveloin-1 cargo, which is associated with malignancy in melanoma patients [47].

**Figure 3:**
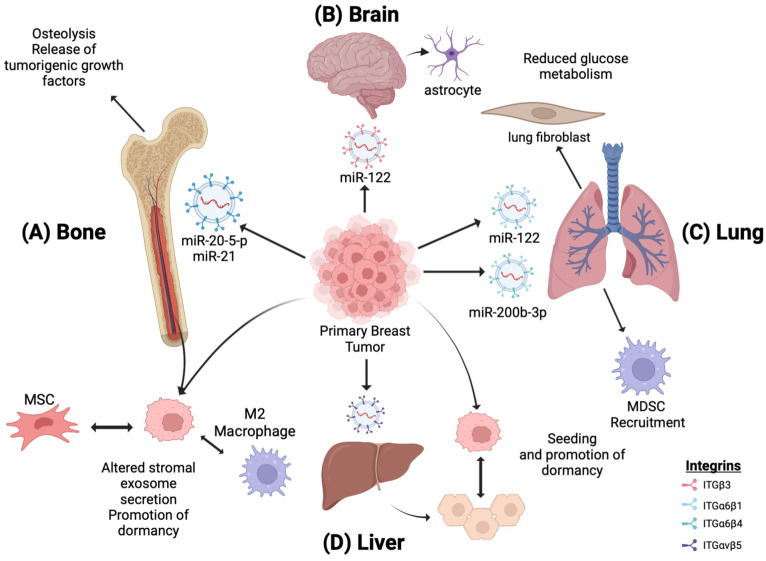
Tumor-derived exosome-induced tissue tropism by miR regulation. EVs secreted by primary breast tumors migrate to distal sites, priming them for breast cancer metastases. (**A**) miR-20-5-p and MiR-21-loaded EVs promote osteoclastogenesis, osteolysis, and metastatic bone invasion. (**B**,**C**) miR-122 downregulates the glucose uptake and metabolism in astrocytes and lung fibroblasts, creating a metabolically favorable environment for Warburg-induced tumor cells. (**C**) miR-200b-3p is taken up by alveolar epithelial type II cells in the lungs, leading to the recruitment of MDSCs and immunosuppressive macrophages and establishing an immunosuppressive premetastatic niche. (**D**) In the liver, primary breast EVs induce changes in the exosomal secretion of resident hepatocytes, promoting metastatic cancer seeding. (**A**,**D**) Additionally, disseminated breast cancer cells promote dormancy by altering the exosome secretion of residential bone marrow and liver cells, respectively.

One approach executed by primary tumor exosomes to promote metastasis involves altering the pre-metastatic niche metabolic landscape by making it more hospitable to Warburg-like tumor cell colonization. Circulating miR-122, a regulator of pyruvate kinase expression, was identified as a marker for metastatic progression in early-stage breast cancer. Upon intravenous injection, breast cancer-derived exosomes containing miR-122 were taken up by lung fibroblasts and brain astrocytes, causing decreased pyruvate kinase and *GLUT1* expression with diminished glucose uptake [48]. The subsequent intracardiac injection of metastatic breast cancer cells led to increased colonization compared to no colonization in mice without exosome treatment (Figure 3A,B). There is a possibility that primary tumor cells sense metabolic changes in the pre-metastatic niche as a trigger to begin metastatic dissemination. Alternatively, it is possible that metastatic tumor cells merely encounter a positive selection in metabolically favorable environments upon systemic dissemination. Further research is needed to tease out these hypotheses and the involvement of exosomes.

In another study involving metabolic changes in brain metastasis, disseminated breast cancer cells engulfed astrocyte-derived exosomes containing miR19a and subsequently lost the expression of the Phosphatase and tensin homolog (*PTEN*), a tumor-suppressor gene [49]. *PTEN* loss activated the *PI3K* signaling pathway, upregulating aerobic glycolysis over oxidative phosphorylation, creating an anabolic, pro-proliferative state characteristic of Warburg tumors. It remains unclear how astrocytes are initially reprogrammed to secrete these miR19a-containing exosomes by disseminated breast cancer cells or exosomes. Nonetheless, these two studies highlight the metabolic changes within the brain premetastatic niche, promoting Warburg tumor metabolism and facilitating breast cancer metastasis, suggesting that they may be particularly susceptible to Warburg-based therapeutic strategies such as:

1. Targeting glycolytic enzymes preferentially elevated in cancer cells (*GLUT1*, *HKII*, *LDHA*, and *PKM2*).
2. Stunting HIF-1α signaling.
3. Engineering chemo-prodrugs that become active under hypoxic and acidic conditions.

Other studies showed that primary breast cancer cells utilize exosomes to alter bone metabolism to a resorptive state and promote metastasis. Breast cancer cells use exosomes to stimulate bone resorption. Osteolysis enables the release of tumorigenic growth factors from the bone matrix, such as *TGF-ꞵ*, *IGF1*, and bone morphogenetic proteins, creating a more favorable environment for metastasis. Exosomes containing miR-20a-5p secreted by TNBC cells promote the proliferation and differentiation of pre-osteoclasts by inhibiting SRC kinase signaling [50]. Additionally, the orthotopic implantation of *SCP28*, a bone metastatic MDA-MB-231 subline, induces the loss of trabecular bone density in mice. A 21-day priming with *SCP28* exosomes increased the tumor burden in the hind limbs of mice and accelerated bone metastasis. Mechanistically, miR-21 was identified within the *SCP28* exosomes as an osteoclast promoter by inhibiting programmed cell death-4 protein function (Figure 3C). These findings suggest that distal exosome secretion from primary breast tumors can induce osteoclast activity in the BM, thereby driving osteolysis and creating a microenvironment that promotes metastasis [51].

In addition to driving metabolic changes that alter the secondary sites for tumor cell dissemination, breast cancer exosomes also modulate the immune landscape of the pre-metastatic niche. For example, Qi et al. showed that Lin28B-expressing tumors secrete exosomes with low let-7s expression, which induces the stemness and migratory capability of the primary tumor. However, upon migration to the lung, exosome-induced neutrophil recruitment and polarization towards an anti-inflammatory N2 phenotype occur, establishing an immunosuppressive niche that facilitates metastasis [52].

Gu et al. observed similar findings in a 4T1 primary breast line. Exosomes isolated from 4T1 primary breast cancer cells were intravenously injected into mice. Upon uptake in the lungs, alveolar epithelial type II cells upregulate the expression of C-C motif chemokine ligand 2 (CCL2), which leads to the recruitment of myeloid-derived suppressor cells and immunosuppressive macrophages, promoting an immunosuppressive, pre-metastatic niche that promoted 4T1 lung colonization. Specifically, miR-200b-3p was identified as the exosome cargo driving CCL2 expression through PTEN inhibition. In these studies, the recruitment of immunosuppressive cells by breast cancer exosomes was suggested as an alternative method of driving lung metastasis [53].

### Tumor Secretome Fosters Metastasis

Cells that migrate from the primary tumor to metastatic sites have received attention, because they promote distant metastases, are used as prognostic markers, and are chemoresistant. Perhaps migratory cells should be considered a part of the tumor’s secretome, since they can efficiently communicate with the circulatory system and tumor microenvironment. Three subtypes of migratory cells are involved in fostering metastasis and relapse and are briefly described below. (A) Cancer stem cells (CSCs) have been isolated from the peripheral blood of cancer patients and are involved in promoting metastases, chemoresistance, and cellular dormancy [54,55]. Furthermore, CSCs can also be viewed as a type of tumor-derived secretome that facilitates metastasis within tumor microenvironments or pre-metastatic niches [56]. (B) Circulating tumor cells (CTCs) isolated from the peripheral blood of cancer patients share phenotypic and functional features similar with CSCs and are a prognostic marker of cancer relapse [57,58]. Some reports have suggested that CTCs leave the primary tumor site as individual cells but cluster together to colonize at metastatic sites in a cooperative manner [59]. However, the size and number of CTCs in the blood can vary, depending on the type of cancer, the stage of the cancer, and the patient’s individual immune system. (C) Disseminating tumor cells (DTCs) have similar characteristics to CSCs, such as the ability to evade the immune system and to resist treatment. However, DTCs also have some unique properties, such as the ability to travel through the bloodstream and to lodge in distant organs. For example, in bone marrow aspirates or lymphatics, DTCs have been isolated and are considered communicative cells that stimulate bone cells to gain entry and colonize [60]. The question of whether CTCs are analogous to CSCs in blood while DTCs are CSCs at metastatic sites remains unknown. Nonetheless, these cells have been implemented in promoting metastasis, and further studies are warranted to decipher the mechanism used by CSCs for colonization as a potential strategy to inhibit metastasis, as well as to elucidate if they secrete exosomes to aid them as they travel through the blood.

## 3. Lymphatic-Induced Tumor Cell Dissemination by Exosome Secretion

The lymphatic system plays a critical role in immune cell tolerance. Given that secreted factors drain into the lymph before dilution into the blood, lymph fluid is thought to be enriched in cancer biomarkers. It is also postulated that cancer-derived exosomes can promote immune cell tolerance by reprogramming T cells in the lymph fluid [61]. 

Several groups, including Broggi et al., identified and characterized exosomes in the lymphatic system of melanoma patients. They demonstrated that melanoma cells could reprogram resident immune cells in the lymph to protect against degradation or an immune response. Lymph collected from melanoma patients undergoing lymph node dissection was enriched in exosomes containing melanoma proteins and miRNA compared to plasma. Distinct proteomic signatures were identified in patients with extra-nodal spread compared to patients with sentinel lymph node proteome. Notably, the exosomes from the extra-nodal spread were enriched with S100 proteins, a well-characterized melanoma antigen previously shown to promote pre-metastatic niche formation [62]. 

**Figure 4:**
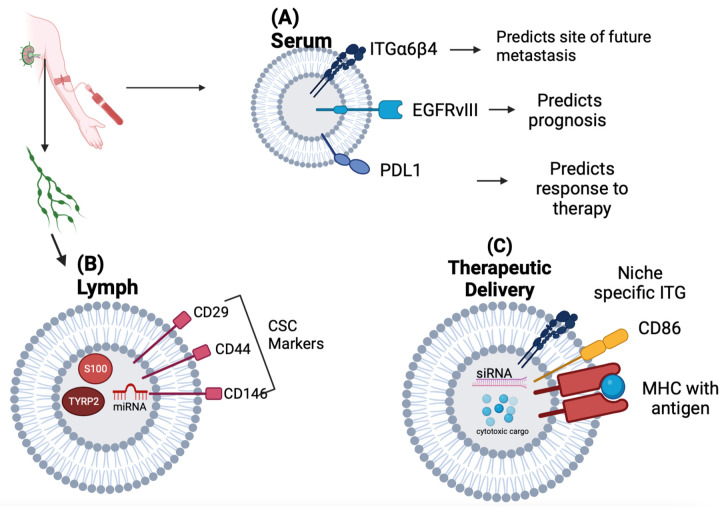
Summary of potential exosome diagnostic and therapeutic applications schematic. (**A**) Exosome surface markers can be detected in patient serum, providing insights on the prognosis and response to treatment. The identification and quantification of the expression of organotrophic integrins can predict sites of current or future metastases (lung metastasis is associated with increased serum ITGβ_4_ and liver metastasis with increased *ITGα*v). The exosome expression of pathogenic receptor EGFRvIII may stratify a subset of glioblastoma patients with a more aggressive disease. The serum levels of exosome PD-L1 may predict the response to chemotherapy and overall survival in lung cancer patients. (**B**) Lymph collected during axillary lymph node dissection can be an abundant source of tumor-derived exosomes and may be helpful in stratifying patients with increased metastasis and a more aggressive disease. Melanoma tumor-derived exosomes in patients with extra-nodal spread beyond the sentinel lymph node have been shown to be enriched in melanoma-specific miRNAs, S100, and TYRP2, among other melanoma-specific proteins. Exosomes from the lymph of breast cancer patients have been shown to express cancer stem cell markers CD29, CD44, and CD146. (**C**) Designing synthetic exosomes embedded with organotrophic integrins may be an intriguing strategy in delivering drug cargos to pre-metastatic niches. Therapeutic exosomes can be loaded with antisense oligonucleotides targeting oncoproteins or oncogenic miRNAs, as well as small molecule cytotoxic agents, to counter p-glycoprotein efflux pumps on tumor cells. Dendritic cell-derived exosomes expressing MHC and costimulatory domains can induce antigen-specific immune responses against tumors.

A study by Ekstrom et al. isolated exosomes from the lymphatic drainage of breast cancer patients during axillary lymph node dissection and identified a subset of markers that contained CSC markers and markers associated with platelets [63]. Overall, the studies from both groups provide evidence that the lymphatic system is an additional source for exosome transport that seems to be involved in promoting metastasis (Figure 4B). Their findings can be extrapolated as biomarkers of tumor progression.

### 3.1. Challenges of Managing Metastatic Disease

Some challenges associated with diagnosing and treating metastatic disease are that it can occur before a diagnosis and undergo cellular dormancy before the primary tumor is detected, as well as the difficulty treating dormant cells [64]. Skeletal metastasis of unknown primary (SMUP) is a condition in which cancer cells have spread from an unknown primary tumor to the bones and presents a new challenge in diagnosing and treating this type of metastasis. This is a rare condition, accounting for 2% of all cancers. The most common sites of metastases are the spine, ribs, pelvis, and skull, which are associated with increased skeletal risk [65]. In a retrospective study of 286 SMUP patients after their initial visit, about 89% of the patients’ tumor origins were detected. Using either blood, a bone biopsy, or CT scans, tumors in the bone were diagnosed with primary tumors of origin as lung cancers (25%), multiple myeloma as the second-leading cancer identified, and about 4% were from breast cancer via blood draws and CT scans [66,67]. SMUP presents a clinical challenge due to the lack of standardized diagnostic tools, complete understanding of the underlying mechanism that causes SMUP, and incomplete clinical data; therefore, there is a need for diagnostic and therapeutic strategies to treat patients. The role of exosomes and the presence of tumor secretome such as CTCs are understudied in SMUP. In both cases, they may provide insight into tumor origins. Currently, chemotherapy and radiation therapy are the most common treatments, with surgery as a palliative treatment option, perhaps implementing bone nanoplatforms loaded with bisphosphonates and chemotherapy as a treatment option for patients. 

### 3.2. Exosome-Induced Cellular Dormancy

Metastatic-promoting traits are obtained at the invasive front of the primary tumor and appear to be selective for what is most advantageous for tumor progression at both local and distant sites [68]. It was suggested by Bainer et al. that primary breast tumor cells reprogram local stromal cells by using transcriptional regulation to phenocopy the metastatic state of the tumor, while their nonmetastatic tumor model had an enhanced proliferative state but was not invasive, indicating that transcriptional changes differ based on the metastatic status of the tumor [69]. Disseminated CSCs can exist in a quiescent state in the BM for decades, evading chemotherapy and endogenous immune responses. Mesenchymal stem cells (MSCs) that surround the central sinus have been shown to control CSC entry into the BM and traffic them towards the endosteum, where they undergo cellular dormancy by forming fap junctions with the bone marrow stroma [70]. An exchange of exosome cargo is involved with the recruitment and reprogramming of MSCs into an immunosuppressive phenotype to induce CSC dormancy. Upon priming, MSCs release exosomes containing miR-222/223, which cause cellular quiescence and P glycoprotein expression when taken up by B-CSCs [15]. Developing neoadjuvant therapies that promote resurgence could subject dormant cells to treatment when undergoing chemotherapy and should be further studied.

Subsequent studies have also supported this phenomenon of MSC priming by using metastatic human breast cell lines that secrete exosomes containing miR-23b to induce cellular dormancy chemoresistance by the downregulation of MARCKS, a driver of cell proliferation and motility [71]. Both studies demonstrated how metastatic cancer cells can prime MSCs to secrete exosomes that promote breast cancer dormancy and chemoresistance. Macrophages are critical in modulating breast cancer dormancy within the bone marrow. For instance, immunosuppressive M2 macrophages promote B-CSC quiescence through gap junctional intercellular communication. The lipopolysaccharide stimulation of TLR 4 on MSCs leads to the recruitment and reprogramming of M2 macrophages. M2 macrophages are then converted from an anti-inflammatory to a proinflammatory M1 phenotype. Once transformed into M1 macrophages, they have been shown to secrete exosomes, driving B-CSC migration and enhanced cell cycling via NF-kB activation. Utilizing macrophage polarization to reverse breast cancer dormancy shows potential as a therapeutic adjuvant to chemotherapy. The delivery of M1 macrophage as an adjuvant to carboplatin in vivo leads to increased B-CSC sensitivity to carboplatin and improved mouse survival [72]. 

Breast cancer dormancy interactions between disseminated metastatic cells and the resident stroma have also been observed in liver metastasis. In a 2D transwell assay, hepatocyte preincubation with MDA-MB-231 primary tumor exosomes increased cancer cell migration and survival. However, in a subsequent 3D human liver micro-physiological system, hepatic milieu preincubation with tumor exosomes increased cancer cell seeding, and hepatic cell proliferation was inhibited. Exosomal miRNA and protein analysis revealed that tumor exosome incubation altered the hepatic niche’s exosomal profile. In addition, the isolation of hepatic niche exosomes and subsequent incubation with aggressive metastatic lines led to the increased expression of E-cadherin, an indicator of a mesenchymal-to-epithelial transition in these cells [73]. This altered secretion promotes metastatic breast cancer cell seeding within the hepatic niche while inhibiting their proliferation and driving a mesenchymal-to-epithelial transition toward cellular dormancy (Figure 3D).

## 4. A Clinical Perspective on Exosomes

For determining whether tumors are present, noninvasive sampling methods such as liquid biopsies are used to isolate tumor-derived exosomes from bodily fluids such as blood, lymphatics, and urine. Circulating exosomes may detect the regression, progression, and recurrence of disease processes. For instance, in bladder cancer, exosomes isolated from urine sub as a liquid easily accessible and consistent with the clinical manifestation of this tumor. Carbonic anhydrase (CA-9) is overexpressed in bladder cancer and is considered a molecular biomarker of this disease. Wen et al. detected CA-9 mRNA in exosomes from 168 bladder cancer patients compared to 90 control patients, with a sensitivity of 85% and specificity of 83% [74], thereby highlighting the versatility of exosome isolation and the reflection of the underlying pathology.

### 4.1. Exosomes as Biomarkers of Tumor Progression

The optimal biomarkers have high specificity for disease and allow for early detection to promote prompt intervention. However, biomarker development is limited by the dichotomy that, while protein-rich bodily fluids may contain disease-specific markers, they are minimal and difficult to detect. In contrast, biomarkers found in more advanced diseases are abundant but are no longer necessary for early-stage detection and interventions. Exosomes offer a more organized and tissue-specific approach to biomarker development based on the intricate packaging and secretion process [75].

Multiple studies have shown the value of exosomes in monitoring resistance to therapy. The mechanisms by which this occurs may vary. Previous works from Corcoran and others provide evidence that docetaxel resistance is directly transferable by exosomes using the multidrug resistance-1 protein, known to efflux taxanes out of the cell [76,77]. Conversely, other studies have reported that the exosome transport of microRNAs promotes post-transcriptional modifications that support tumor progression. One study showed that exposure to 5-FU increased microRNA secretion in colon cancer cells, resulting in altered intracellular physiology and resistance to treatment [78], while another study also uncovered that microRNA transport through exosomes decreased the expression of CyclinD1 in glioblastoma cells, resulting in cellular quiescence and chemoresistance [79]. 

Serum exosomes may show promise as biomarkers of cancer prognosis compared to bone marrow aspirates commonly used in clinics to determine bone marrow metastasis. Lung cancer patients who showed a response to chemotherapy and overall improved survival were noted to have lower levels of *PD-L1*-derived exosomes [80]. Studies have also shown that pathogenic receptor *EGFRvIII* may be “shared” among glioblastoma cells through exosomes. In this context, Skog et al. confirmed that exosomes might allow for the secretion of pathogenic receptor *EGFRvII*I and are readily detected in the serum of patients with glioblastoma [81]. Thus, exosome proteins such as *PD-L1* and *EGFRvIII* may serve as plasma markers for cancer prognosis. In addition to serving as direct biomarkers of diseases, it is now evident that exosomes isolated from body fluids can be analyzed for their donor and recipient cell types based on their cargos and surface markers (Figure 4A). Finally, exosomal integrins can also serve as biomarkers to predict metastasis [82]. Regardless of the tumor type, patients with lung metastasis were found to have elevated levels of exosome integrin β4 compared to those with liver or no metastasis. Patients with liver metastasis had higher levels of exosomal integrin αv than those with lung or no metastasis. Pancreatic cancer patients that went on to develop liver metastasis had the highest levels of integrin αv at the time of diagnosis.

In summary, exosomes from various bodily fluids may serve as functional biomarkers for cancer detection, resistance to therapy, and disease progression. In addition, exosome isolation from bodily fluids is a minimally invasive method to assess pathology and malignancy mechanisms. Further development for GLP grade isolation and characterization markers will enable us to adopt isolation techniques for clinical application.

### 4.2. Exosomes as Delivery Systems

In the study by Kamerkar et al., transfected exosomes with a siRNA targeting an oncogenic *KRAS G12D* mutant in pancreatic ductal adenocarcinoma [83]. Upon repeated intraperitoneal injections in mice with orthotopic PDAC tumors, the exosomes displayed staunch retention in the bloodstream, with effective tumor uptake, reduced *KRAS G12D* mRNA levels, impaired tumor growth, and improved survival over 30 days. This technology is currently being utilized in a phase I clinical trial on patients with advanced stage PDAC at MD Anderson (NCT03608631) (Table 1).

In addition to delivering antisense oligonucleotides, exosomes can be loaded with small molecule chemotherapeutics. For example, macrophage-derived exosomes loaded with paclitaxel through sonication were incubated with a malignant canine kidney cell line expressing P-glycoprotein. The loaded exosomes displayed enhanced cytotoxicity compared to free paclitaxel in both drug resistance and sensitivity [84]. Furthermore, in a P-glycoprotein-positive murine model of pulmonary metastasis, exosome-loaded paclitaxel therapy conferred reduced tumor growth compared to free paclitaxel (Figure 4C).

Cancer vaccination strategies utilizing exosomes loaded with antigenic peptides have been evaluated in animal and clinical trials [85]. Dendritic cell-derived exosomes have received attention because they express MHC I and II and CD86 and can activate CD4+ and CD8+ T-cell responses. It has been demonstrated that dendritic exosomes can induce antigen-specific immune responses several ways, including endocytosis and the internalization of antigenic peptides on dendritic cells. These peptides are then displayed by surface MHC molecules, the direct transfer of MHC/antigen complexes to the dendritic cell surface, and the direct transfer of MHC/antigen complexes to the tumor cell surface, the latter triggering direct T cell-mediated tumor targeting [86]. In a phase I study, a treatment with autologous dendritic cell exosomes loaded with genes from melanoma tumor antigens was well tolerated in non-small cell lung cancer patients. In a subset of these patients, antigen-specific T-cell activation was also observed [87]. Building from this work, it has been found that stimulating dendritic cells with IFN-γ prior to exosome collection triggers increased T- and NK-cell responses in vitro and may confer a more immunogenic vaccine [88]. In a phase II clinical trial (NCT01159288), allogeneic IFN-γ mature Dex loaded with MHC-restricted cancer antigens was administered as maintenance immunotherapy in 22 patients with unresectable NSCLC. Dex therapy enhanced the NK-cell responses, and seven patients had tumor progression stabilization for >4 months. Additionally, in a phase I/II study in Japan, SART1-pulsed Dex enhanced cytotoxic T-cell responses in patients with advanced squamous cell carcinoma of the esophagus [89].

**Table 1:** A clinical perspective on exosomes.

	Exosomal Biomarker	Significance		Reference
Serum	PDL1	Correlation with Tumor PDL1 expression, Response to Immunotherapy, and Overall Survival in NSCLC		[69]
	EGFRvIII mRNA	Able to identify mutation status in serum via RT-PCR to predict prognosis and identify therapy options (vaccine vs tyrosine kinase inhibitor) in Glioblastoma Multiforme		[70]
	ITGβ_4_	Associated with Lung Metastasis in Breast Cancer		[42]
	ITGα_v_	Associated with Liver Metastasis in Breast and Pancreatic Cancer Predictor of Liver Metastasis in Pancreatic Cancer		
Lymphatics	S100 TYRP2	Associated with extra-nodal spread in melanoma patients undergoing lymph node dissection		[73]
	CSC Markers (CD 29, CD44, CD146)	Identified in Breast Cancer patients undergoing axillary lymph node dissection		[74]
Therapeutic Delivery		**Approach**	**Stage**	**Reference**
	Anti-Sense Oligonucleotides	KRAS G12D siRNA For Pancreatic Ductal Adenocarcinoma	Phase I Trial: NCT03608631	[75]
		Anti-miR-9 for drug resistant Glioblastoma Multiforme	In-vitro	[80]
	Chemotherapeutic Loading	Paclitaxel-loaded macrophage-derived exosomes in G-Glycoprotein expressing Cancers	In-Vivo murine model	[76]
	Dendritic Cell Derived exosome (DEX) based Cancer Vaccination Strategies	Autologous DEX-loaded with MAGE antigens in NSCLC	Phase I Trial	[78]
		Allogeneic Dex pulsed with INF-Y-loaded with MHC class I- and class II-restricted cancer antigens for maintenance immunotherapy in NSCLC	Phase II Trial: NCT01159288	[87]
		Dex-derived from DCs pulsed with SART1 for advanced squamous cell carcinoma of the esophagus	Phase I/II Trial	[88]

Identifying the genetic material and its use in promoting cellular reprogramming provides potential therapeutic strategies to reduce metastasis [90,91]. To achieve successful treatments, it is essential to target the desired cells or tissues efficiently, avoid off-target effects, and ensure stability and reproducibility while overcoming barriers such as the immune system and the blood–brain barrier.

Great strides have been made to overcome some of the challenges associated with immune cell activation, including engineering exosomes with immune-suppressing agents such as “self” antigens or coating them with molecules that prevent immunogenicity to increase their delivery to recipient cells [92,93]. Additionally, viral nanoparticles are nanocarriers with modified viral capsids that can deliver genetic material such as siRNA or mRNA to cells without invoking an immune response due to their high permeability specificity [94]. Comprehensive studies are necessary to assess the safety and immunogenicity of exosome-based therapies or viral nanoparticles. This includes investigating potential immune responses, evaluating long-term effects, and identifying strategies to mitigate immunogenic reactions. 

Targeting glioblastoma has been challenging due to the blood–brain barrier. Munoz et al. used MSC-mediated delivery to inhibit TMZ resistance [90], demonstrating that MSCs could cross the BBB and circumvent the bioaccumulation of nascent exosomes, which normally accumulate in the liver or spleen after injection. Others have engineered exosomes with modified surface proteins that are similar to proteins found in brain cells. Other strategies include fusing exosomes to liposomes as a delivery system to the brain [95]. Whether using cells or synthetic exosomes to cross the blood–brain barrier, the size and composition must be small enough to traverse this area. In addition, the use of nanotechnology-based approaches such as liposomes, nanoparticles, and viral vectors can enhance the delivery of exosomes across the blood–brain barrier or other biological barriers.

### 4.3. Nanotechnology-Based Approaches

Nanocarriers are a type of nanotechnology that is used to deliver drugs or therapeutic agents in a targeted manner and can be engineered to have specific properties, such as size, shape, surface charge, and functional groups, that can enhance their stability, solubility, and targeting ability. Nanocarriers are typically engineered to have specific properties that allow them to encapsulate or attach therapeutic agents and transport them to the desired site in the body. Furthermore, nanocarriers may provide solutions to rectify off-target effects and overcome immune system and blood–brain barriers that thwart clinical exosome applications. For example, core–shell nanocarriers containing miRNA coupled with docetaxel were used as codelivery systems in treating metastatic breast cancer, and miR34a encapsulated in the core of the nanocarrier was shielded from RNase degradation, while the shell interacted with the caveolae-mediated pathway to prevent lysosome degradation [96]. 

Types of nanocarriers include liposomes, polymeric nanoparticles, dendrimers, and micelles [97]. Strategies based on inhibiting or replacing miRs have emerged as promising approaches in cancer therapy, given that miRs can act as either oncogenes or tumor suppressors. Exosomes have nanocarrier-like properties and are being used in research to deliver anti-miR, chemotherapy, or ATP molecules for cancer treatment and chemoresistance [84,98,99,100]. For example, *MRX34* is a liposome-encapsulated miRNA that targets tumor suppressors miRNA, miR21, and miR-21 and is overexpressed in many types of cancer, and it plays a role in promoting tumor growth and metastasis. *MRX34* works by downregulating miR-21 expression, thereby inhibiting tumor growth and metastasis. *MRX34* is relatively safe and is well tolerated in patients. There was a phase I/II trial on pancreatic cancer where *MRX34* was shown to reduce tumor sizes in some patients [101]. Further, *MRX34* coupled with dexamethasone pretreatment reduced tumor sizes and improved cancer patient outcomes in seven cancer types, including solid tumors and hematology malignancy [102]. 

Desantis et al. specifically provided an overview of three different miR-based nanocarriers, liposomes, polymers, and exosomes, and highlighted their potential as codelivery molecules that were used to increase stability and decrease degradation. Further, they described various nanoparticle systems that incorporated fungoides, non-living bacteria, or locked nucleic acid oligonucleotides complexes with anti-miR or mimic miR responses that have been implemented to treat various types of cancer, including multiple myeloma [103]. Although nanocarriers offer precise targeted therapies with increased efficacy, such as miR stability for treating cancer patients, a small percentage of nanocarriers are approved for clinical application [104]. In addition to liposomes and other biomaterials such as dendrimers, PEGs were shown to overcome some clinical barriers such as interpatient and tumor heterogeneity and immune cells and renal clearance, as well as bioaccumulation [105]; however, the microenvironment and bioavailability must be considered when designing personalized treatments. 

Exosomes have been modified and engineered in several ways to deliver targeted drugs, including altering exosome surfaces with specific ligands, vaccines, or antibodies to enhance tumor cell targeting [106,107]. Additionally, loading techniques such as electroporation, sonication, and lipid-based approaches have been explored to improve the loading capacity of exosomes with therapeutic cargos. 

Engineered exosomes coupled to liposomes, PEGs, or other molecules have also been reported to increase exosome drug delivery in a controllable way and could circumvent pharmacokinetic drawbacks [108]. Poly(amidoamine) PAMAM dendrimers can be utilized as a loading technique for exosomes to achieve the encapsulation or attachment of specific cargo molecules. The study from Nair et al. used the PAMAM dendrimer–exosome hybrid approach to increase the delivery of siRNA and *PD-L1* cargo to cells with greater efficacy than dendrimers alone, which seemed to be exosome-driven, thereby evading the concerns associated with exosome transport or premature degradation before reaching its target [109]. 

Nanocarriers can address some challenges associated with exosome therapeutic potential. For example, encapsulating exosomes in nanocarriers would provide a universal size distribution, and their cargo composition would also be uniform. Additionally, adding surface modifications to exosome-loaded nanocarriers could increase the exosome circulation time and biodistribution. Finally, given that nanocarriers can be synthesized and manufactured in large batches, identifying exosome cargo and loading them into nanocarriers could address the concerns regarding the variations in the isolation methods or exosome heterogeneity.

## 5. Challenges and Drawbacks to Clinical Application of Exosomes

While exosomes hold promise for various therapeutic applications, they also present several disadvantages when treating cancer patients. Here are some of the key drawbacks:

1. Exosome heterogeneity is thought to be a key factor in determining their function and effectiveness in intercellular communication. By displaying different properties and compositions, exosomes can interact with different target cells and activate different signaling pathways [110]. Moreover, the cells that secrete exosomes can also be different between individuals, making it difficult to identify the source of exosome heterogeneity, especially in cancer, where cancer cells themselves are heterogenous [111,112]. Efforts have been made to develop standardized protocols for exosome isolation, purification, and characterization. These protocols aim to ensure the consistent quality and purity of exosome preparations, reducing the heterogeneity and allowing for more reliable and reproducible therapeutic applications. However, the complexity of exosome heterogeneity, removal of undesirable contaminants, and different isolation methods make it difficult to standardize the procedures.
2. A consensus is needed for acceptable purification standards that are well tolerated with a maximum payload. Comparisons of various isolation steps across different cell lines or ex vivo samples such as urine and blood are currently underway [113,114,115]; however, the samples are normally collected in a laboratory setting without consideration for good manufacturing practices or differences in storage conditions, which also need to be standardized.
3. Exosomes have limited cargo capacity due to their size, which affects how much and what type of therapeutic agents can be loaded into them, as well as their delivery speed and clearance rate [116]. Furthermore, because exosomes are released from cells in a variety of sizes, shapes, and compositions, it is difficult to ensure a consistent therapeutic effect when using them as targets. Coating exosomes with stealth materials such as polyethylene glycol (PEG) or using a nanocarrier can increase an exosome’s circulation time in the bloodstream.
4. Targeting exosomes at breast cancer cells specifically can be challenging without proper selective markers, since exosomes are distributed throughout the body and potentially interact with healthy tissues, leading to off-target effects and reduced therapeutic efficiency. Additionally, exosome instability can also be problematic for cancer treatment, as their components can undergo rapid changes when exposed to different environmental conditions. Exosomes can degrade quickly, making them difficult to transport and store for long periods of time. Perhaps synthesizing exosomes from the same cell type will have the same surface proteins that are recognized and engulfed by the recipient cell. Additionally, using nanocarrier systems will protect exosomes from degradation and enhance their delivery to specific target cells.
5. The large-scale production of exosomes for clinical use can be technically challenging and costly. Standardized manufacturing processes need to be established to ensure the consistent quality, purity, and potency of exosome-based therapeutics. Scaling up production while maintaining batch-to-batch consistency remains a significant hurdle. Moreover, there is a need for standardized methods for the isolation, characterization, and quantification of exosomes to ensure consistency and reproducibility in their use as therapeutic agents.
6. As with any novel therapeutic approach, exosome-based treatments must undergo rigorous testing and regulatory approval processes. Regulatory challenges associated with exosome clinical implementation that include characterization, standardization, and administration are currently under review [117]. Ensuring exosome-based delivery system safety and efficacy, along with addressing concerns regarding biodistribution and bioaccumulation as potential long-term side effects, is crucial to elucidate before widespread clinical implementation can occur. Further, regulatory agencies, including the FDA and European Medicine Agency, are actively working to establish guidelines and frameworks specifically tailored to exosome-based therapies [118,119]. Although these advances are promising in addressing exosome therapy limitations, additional research and clinical trials are needed to validate their effectiveness and safety.

## 6. Discussion

Three types of tumor-derived extracellular vesicles are reported to promote cancer metastasis and were discussed in our review, with exosomes as the primary focus. Several proteins overlap between the TD-Evs, regardless of how they are synthesized. Yet, the degree to which these proteins are expressed varies and is regulated by the donor cell. Interestingly, exosomes mainly contain RNA-processing biomolecules, while the other two vesicles contain antigens or plasma proteins that drive rapid responses in the recipient cells by transferring membrane proteins [25,31]. Despite sharing similar protein markers, all three TD-Evs seem to facilitate tumor progression differently, which is predicated on their respective biogenesis and their contents. Although exosomes have been studied more than the other subtypes as drug delivery systems, biomarkers, or targets, they should also be studied as possible delivery systems. It remains unclear what triggers TD-EV secretion and whether tumor cells secrete them in a stochastic or non-stochastic manner is still elusive. Elucidating the mechanisms that trigger this response will assist us in how we efficiently and safely deliver TD-Evs to the designated area.

Several clinical trials are currently underway to test the safety and efficacy of miRNAs in treating breast cancer. Some of these trials are evaluating the use of miRNAs alone, while others are evaluating the use of miRNAs in combination with other cancer therapies, ultimately choosing which miR nanocarriers will need to consider the microenvironment and bioavailability of the particle to ensure that the miR payload is delivered to the intended cell or tissue. 

The complexity of tumor progression demands a multi-level therapeutic strategy to inhibit metastasis, which has ushered in a new wave of cancer therapies and diagnostic tools for cancer progression, including exosomes as drug delivery systems. The synergistic effects of combining exosome therapy with other treatment modalities, such as chemotherapy, radiation therapy, or immunotherapy using nanocarriers as delivery vehicles, has proven to be effective in preclinical models, while tumor and interpatient heterogeneity pose challenges to implementing nanomedicines in patients [84,99,100]. 

Some approaches include loading exosomes with therapeutic molecules by incubating them with the desired cargo via chemical manipulation, such as diffusion, electrophoresis, or sonication, and then delivering drugs to cancer cells [120,121]. Other studies have used bioengineering techniques to modify exosomes for enhanced therapeutic efficacy. This includes the incorporation of specific proteins or peptides onto exosome surfaces to improve their targeting capabilities [122], as well as genetic modifications of exosome-producing cells [123]. The caveat is that most of these studies were performed using cell lines or mouse models; thus, further studies are warranted to optimize the loading capacity of exosomes with drugs, and their administration and biodistribution should be assessed clinically. 

Several studies have investigated exosomes’ ability to inhibit cancer metastases; only a small subset of delivery systems has been tested in clinical trials, and none have received FDA approval [104,121,124]. This suggests that the drawbacks discussed in our review, such as bioavailability, distribution, and exosome heterogeneity, are concerns that need to be further studied. Other concerns such as scalability, administration, safety standards, and isolation methods need to be optimized as well. Additionally, the isolation methods, good manufacturing practices, safety, and toxicity need to be evaluated. Exosomes are sensitive to various environmental conditions, such as temperature and pH, which can alter exosomal membranes, making them susceptible to degradation, as well as alter their function [125]. The changes in exosome stability also emphasize the need for standardization in how exosomes are stored and should be further studied before they can be administered to patients. Given that PAMAM dendrimers have been shown to be effective in protecting exosomes from a variety of stresses, including heat, pH, and enzymes, they are ideal nanocarriers to shield exosomes from degradation and could be a good “packing material” for exosome storage and transport; however, more research is necessary to fully understand how PAMAM dendrimers maintain exosome stability in different cellular microenvironments, as well as their clinical application.

## 7. Future Perspectives

Given our current understanding of the role of exosome integrins in driving tumor organotropism, it may be fruitful to explore hijacking this process as a therapeutic strategy. As mentioned earlier, integrins A6β4 and αvβ5 were identified in exosomes and established as necessary for lung and liver tropism through shRNA knockdown and antibody neutralization studies. Targeting these integrins may serve to prevent metastasis in high-risk patients. Additionally, designing synthetic exosomes embedded with these integrins may be a strategy for delivering drug cargos to metastatic sites.

Furthermore, integrins in exosomes can also be predictors of the metastatic propensity and future sites of metastasis across tumor types. This may have significant clinical benefits in stratifying cancer surveillance, leading to earlier screening during remission and the early initiation of relapse chemotherapy, as well as more accurate prognostics for care decision-making goals. However, future works are needed to help develop and standardize integrins as a cancer biomarker.

Exosomes have tremendous potential in cancer drug delivery due to their low immunogenicity, low cellular toxicity, and preferential accumulation in tumor tissues over normal tissues [126]. However, optimization is needed to improve production and standardization to develop exosome-based therapeutics. Notably, the current production protocols have a meager exosome yield due to the high cost of cultures and inefficiencies in isolation and purification. Several groups are working to optimize the output by manipulating gene expressions and culture conditions, notably by overexpressing heat shock protein 20, inducing hypoxia and inflammation, increasing intracellular calcium, and utilizing 3D culture-based systems [127]. Microfluidics-based manufacturing offers a promising approach for the scalable and controlled production of exosomes. It allows for the precise manipulation of fluids and particles on a microscale, enabling the high-throughput production of homogeneous exosome populations, with improved yield and reproducibility [128].

Additionally, exosomes have poor cargo loading and size standardization, leading to a potentially unpredictable biodistribution; a technique currently being investigated to standardize the size involves using semi-continuous size exclusion chromatography [129]. 

## 8. Conclusions

The pliancy of exosomes makes them a promising target for therapeutic intervention. Exosome sizes allow them to easily travel through the bloodstream or enter cells via gap junctions. Their lipid bilayer is also pliable and can be modified to deliver cargos that include crossing the blood–brain barrier. Finally, exosome cargos alter cell functions by regulating gene expression, protein synthesis, and apoptosis; therefore making them an optimal drug delivery system or optimal for developing strategies that target cancer metastasis and chemoresistance. As the field optimizes scalability and administration, while addressing safety and toxicity, nanocarriers will become a game changer for how exosome-based therapies are delivered to cancer patients.

## Data Availability

Not applicable.
